# A systematic review of the impact of antifungal stewardship interventions in the United States

**DOI:** 10.1186/s12941-019-0323-z

**Published:** 2019-08-21

**Authors:** Emily Hart, Melanie Nguyen, Meghan Allen, Collin M. Clark, David M. Jacobs

**Affiliations:** Department of Pharmacy Practice, School of Pharmacy and Pharmaceutical Sciences, University at Buffalo, 316 Pharmacy Building, Buffalo, NY USA

**Keywords:** Antifungal stewardship, Antimicrobial stewardship, Invasive fungal disease, Antifungal agents

## Abstract

**Background:**

Antimicrobial resistance is a widely recognized public health threat, and stewardship interventions to combat this problem are well described. Less is known about antifungal stewardship (AFS) initiatives and their influence within the United States. The purpose of this study was to evaluate evidence on the impact of AFS interventions on clinical and performance measures.

**Methods:**

A systematic review of English language studies identified in the PubMed and EMBASE databases was performed through November 2017. The review was conducted in accordance with PRISMA. Search terms included antifungal stewardship, antimicrobial stewardship, *Candida*, candidemia, candiduria, and invasive fungal disease. Eligible studies were those that described an AFS program or intervention occurring in the US and evaluated clinical or performance measures.

**Results:**

Fifty-four articles were identified and 13 were included. Five studies evaluated AFS interventions and reported clinical outcomes (mortality and length of stay) and performance measures (appropriate antifungal choice and time to therapy). The remaining eight studies evaluated general stewardship interventions and reported data on antifungal consumption. All studies were single center, quasi-experimental with varying interventions across studies. AFS programs had no impact on mortality (3 of 3 studies), with an overall rate of 27% in the intervention group and 23% in the non-intervention group. Length of stay (5 of 5) was also similar between groups (range, 9–25 vs. 11–22). Time to antifungal therapy improved in 2 of 5 studies, and appropriate choice of antifungal increased in 2 of 2 studies. Antifungal consumption was significantly blunted or reduced following stewardship initiation (8 of 8), although a direct comparison between studies was not possible due to a lack of common units.

**Conclusion:**

The available evidence suggests that AFS interventions can improve performance measures and decrease antifungal consumption. Although this review did not detect improvements in clinical outcomes, significant adverse outcomes were not reported.

## Introduction

Antimicrobial resistance is a growing public health challenge that poses a global threat [1]. In the United States, at least 2 million people acquire and at least 23,000 people die each year from an antibiotic-resistant infection [2]. Approaches to optimize antibiotic use and contain antimicrobial resistance have been recommended to preserve the benefits of antibiotics and provide the best patient care. Antimicrobial stewardship programs (ASPs) have received particular attention because of their focus on improving health outcomes and patient care. The Infectious Diseases Society of America (IDSA) and the Society for Healthcare Epidemiology of America published an updated guideline on the implementation of ASPs within inpatient populations [3]. ASPs are defined as coordinated efforts designed to improve the appropriate use of antibiotics by promoting the selection of the optimal antibiotic regimen [3]. The benefits of ASPs are well documented and include improved patient outcomes, reduced *Clostridium difficile* infections, and optimized resource utilization across the continuum of care [3–8].

Although ASPs have primarily focused on antibiotics, antifungal resistance is a growing and emerging threat [9]. *Candida* infections due to fluconazole- and echinocandin-resistant strains are increasingly prevalent and comprise over 70% of resistant isolates from *Candida glabrata* or *Candida krusei* species [10, 11]. *Candida auris* is also an emerging multi-drug resistant pathogen with cases or outbreaks reported in over 20 countries since its first discovery in 2009 [12]. This is especially concerning given that *C. auris* isolates can be resistant to all three of the main classes of antifungal drugs, and cases can go undetected as it is commonly misidentified in clinical laboratories [12]. Further, although antifungal resistance is common in *Candida* species, emerging threats also include azole-resistant *Aspergillus fumigatus* [13]. Several studies have shown that antifungal agent use can deviate from guidelines and that this has a negative impact on patient outcomes [14–17]. Appropriate antifungal use is an important factor in fighting drug resistance [18]. Given the rise in drug resistance and the documented inappropriate use of antifungals, the implementation of formal antifungal stewardship (AFS) programs is becoming increasingly important.

Antifungal stewardship inherently has different complexities and clinical priorities to antimicrobial stewardship, but ultimately they share a common goal of improving appropriate drug use through regimen optimization [18–20]. AFS programs are emerging as a sub-specialty of ASPs, yet the literature on these programs is sparse. The purpose of this systematic review is to summarize evidence on AFS programs in the United States and evaluate their impact on clinical and performance outcomes. We focus on US-based programs given the differences in healthcare systems and resources around the world (i.e., settings where antifungals are available without a prescription).

## Methods

### Search strategy

A comprehensive search for articles relevant to ASP implementation, with a focus on programs specifically obtaining data on AFS, was conducted through November 2017. Two researchers independently conducted the search, and inclusion was determined based on a consensus of the relevance of the identified study. The literature search was carried out through the PubMed and EMBASE online databases utilizing the following terms: antifungal AND stewardship, antifungal stewardship, antimicrobial stewardship AND candida AND invasive fungal, antimicrobial stewardship AND invasive fungal, antimicrobial stewardship AND candida, antimicrobial stewardship AND candiduria AND invasive fungal, invasive fungal disease AND stewardship, stewardship AND candida AND candidemia AND candiduria, stewardship AND candida, stewardship AND candidemia, stewardship AND candiduria. In addition, to increase completeness the reference lists of relevant articles were searched.

### Inclusion and exclusion criteria

Studies were included if: (a) the article described an AFS program or intervention; (b) the program was implemented in the United States; and (c) the article reported an AFS program containing data on clinical or performance measures. Exclusion criteria were determined according to the following components: (a) not written in English; (b) study did not include an intervention; and (c) did not evaluate an outcome of interest. Outcomes of interest were divided into performance measures (appropriate fungal choice, time to therapy, and antifungal consumption) and clinical measures (mortality and length of stay).

### Study selection and data extraction

Both researchers independently screened all titles and abstracts identified in the literature search. All abstracts were considered if they met the inclusion criteria, and full-text articles were then retrieved for further review. All disagreements over eligibility were resolved via discussion between the researchers.

After obtaining the full-text articles meeting the inclusion criteria, two investigators extracted data using a standardized form that included study title, year of publication, author, objectives, design, patient population, duration, site, intervention description, and findings pertaining to outcomes of interest. An Excel spreadsheet (Microsoft Corp., Redmond, WA) compiling all variables to be extracted was used to ensure data extraction reproducibility and completeness. A second researcher further reviewed the extracted data to verify the necessity for the data. Any disagreements on data inclusion were confirmed through discussion between all the researchers.

### Synthesis of results

The Preferred Reporting Items for Systematic Reviews and Meta-Analyses (PRISMA) checklist was used to guide the systematic review [21]. Due to variability in interventions, patient populations, and outcome measures, extracted data were summarized descriptively. Conclusions were drawn based on qualitative synthesis of the findings.

## Results

### Search results

**Fig. 1:**
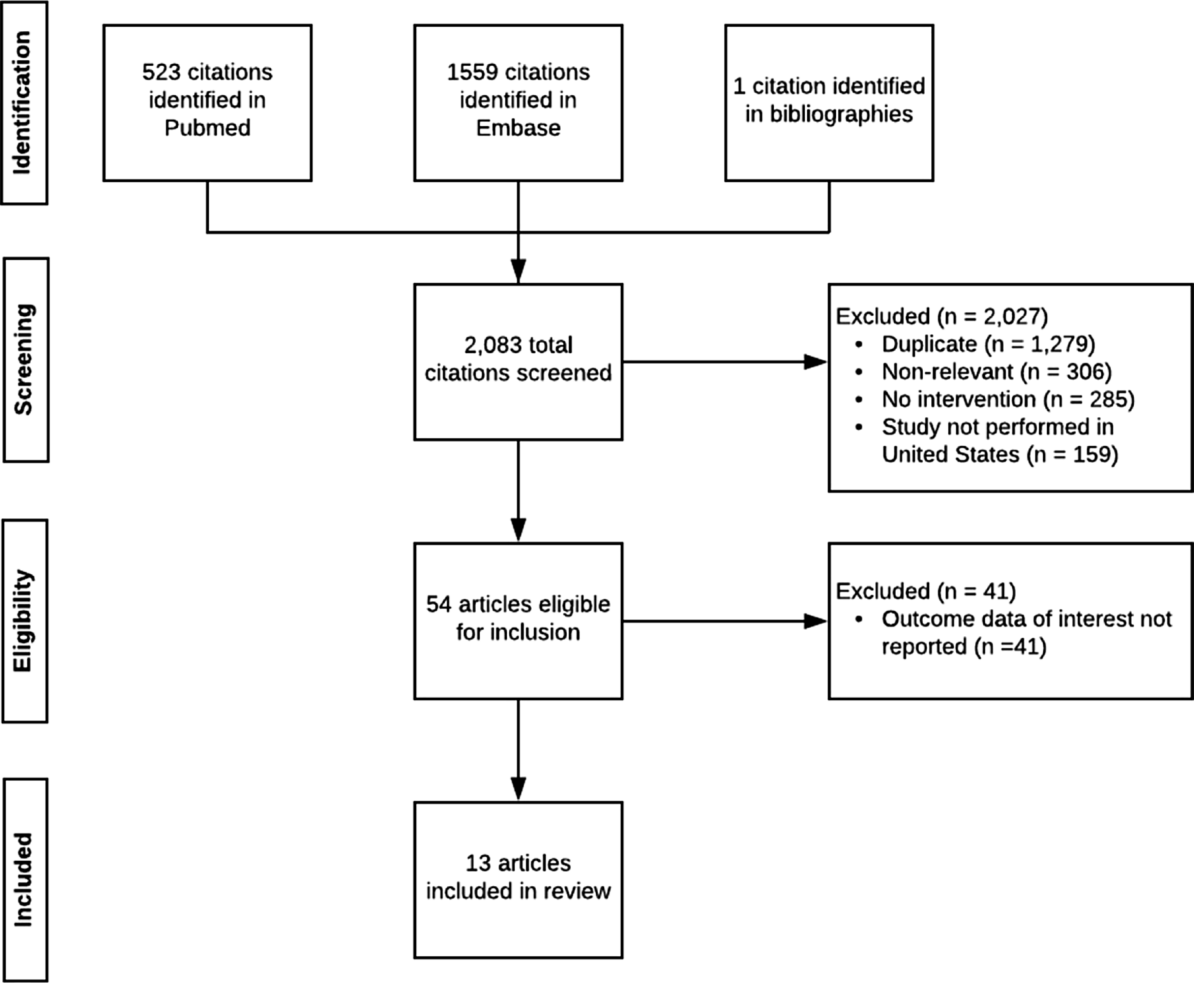
PRISMA flow diagram of the search process and study selection

A total of 2083 studies were initially screened for inclusion by title and abstract. After excluding duplicates, non-relevant studies, non-interventional studies, and studies performed outside the US, 54 articles were eligible for full-text assessment. Of these, 41 did not report data on clinical and performance measures and were excluded. Thus, 13 articles were included in the systematic review (Fig. 1).

### Study characteristics

Of the included articles, five evaluated AFS interventions and reported clinical outcomes (mortality and length of stay) and performance outcomes (appropriate antifungal choice and time to therapy) [22–26]. These studies are summarized in Table 1. The remaining eight studies, summarized in Table 2, evaluated general antimicrobial stewardship interventions and reported data on antifungal consumption [27–34].

**Table 1:** Clinical and performance measures reported within antifungal stewardship interventions

References	Design	Setting and population	Intervention	Number of patients		Outcomes (non-intervention vs. intervention)			
				Non-intervention	Intervention	Mortality	Length of stay (LOS)	Appropriate choice	Time to therapy
Anthworth et al. [22]	Quasi-experimental	930-bed academic hospital Adult and pediatric patients with positive *Candida* blood cultures	Care bundle for candidemia	37	41	Not reported	Overall LOS: 21 days vs. 20 days *NS*	Appropriate empiric therapy: 36 (97.3%) vs. 40 (97.5%) *NS* Appropriate therapy after sensitivity testing: 32 (86.5%) vs. 41 (100%) *Increased, p = 0.0488*	Initiated antifungal therapy within 24 h of positive Gram stain: 33 (89.2%) vs. 38 (92.7%) *NS*
Reed et al. [23]	Quasi-experimental	1229-bed teaching hospital Patients aged 18–89 with positive *Candida* blood cultures	Pharmacist recommendations	85	88	In-hospital mortality: 16 (19%) vs. 26 (30%) *NS*	Overall LOS: 15 days vs. 19 days *NS* Infection-related LOS: 10 days vs. 11 days *NS*	Effective antifungal choice: 67 (88%) vs. 80 (99%) *Increased, p = 0.008*	Median time from Gram stain to effective antifungal hang time: 13.5 h vs. 1.3 h *Decreased, p = 0.04* Median time from Gram stain to effective order placement: 9.2 h vs. 0.1 h *Decreased, p = 0.01*
Heil et al. [24]	Before-and-after	Setting not reported Hospitalized patients with blood cultures positive for *Candida*	Diagnostic tool for *Candida*	61	21	In-hospital mortality: 19 (31%) vs. 5 (24%) *NS*	Median LOS: 25 days vs. 12 days *NS*	Not reported	Mean time to targeted therapy: 2.3 days vs. 0.6 days *Decreased, p = 0.0016* Received targeted therapy after 1 week: 57 (93%) vs. 21 (100%) *NS*
Huang et al. [25]	Pre-post quasi-experimental	University hospital Patients aged 18 and older with bloodstream infections	Diagnostic tool for candida and bacteremia	22	20	30-day mortality (yeast infection): 6/18 (33.3%) vs. 3/17 (17.7%) *NS*	Overall LOS (yeast infection): 20.9 days vs. 22.1 days *NS* ICU LOS (yeast infection): 15.1 vs. 10.3 *NS*	Not reported	Time to effective therapy (yeast infection): 68.6 h vs. 45.6 h *NS* Time to optimal therapy (yeast infection): 57.1 h vs. 50.9 h *NS*
Pardo et al. [26]	Pre-post quasi-experimental	939 bed academic medical center Adult patients with Gram positive bacteremia or candidemia	Diagnostic tool for gram positive bacteria and candida	27	9	In-hospital mortality (*Candida* infection): 5 (19%) vs. 1 (11%) *NS*	Median LOS (*Candida* infection): 9.1 days vs. 11 days *NS* ICU LOS (*Candida* infection): 0 days vs. 0 days *NS*	Not reported	Median time to therapy from culture collection (*Candida* infection): 33.7 h vs. 36.7 h NS Median time to therapy from culture positivity (*Candida* infection): 4.4 h vs. 3.9 h *NS*

*ICU* intensive care unit, *LOS* length of stay, *NS* not significant

**Table 2:** Antifungal consumption reported within general antimicrobial stewardship interventions

References	Design	Setting	Population	Intervention	Utilization				
					Drug/class	Unit of Measurement	Non-intervention	Intervention	*p*
Storey et al. [27]	Quasi-experimental	100-bed community hospital	All inpatients on medical/surgical service who received more than 2 days of antimicrobial therapy	Audit and feedback twice weekly	Antifungals	DDD per 100 admissions	23.1	13.2	*0.035*
						DDD per 1000 PD	59.1	36.5	*0.047*
					Echinocandins	DDD per 100 admissions	4.7	3.2	0.975
						DDD per 1000 PD	11.3	8.7	0.924
					Fluconazole	DDD per 100 admissions	17.6	9.8	0.105
						DDD per 1000 PD	46.1	27.2	0.284
Cook et al. [28]	Quasi-experimental	904-bed tertiary care teaching hospital	Patients who received controlled or restricted antimicrobials	48-h review for controlled antimicrobials, restrictions and preauthorization	Antifungals	DDD per 1000 PD	151.9	44	*< .0001*
Jenkins et al. [29]	Quasi-experimental, interrupted time series	525-bed public safety-net hospital	Any patient receiving antimicrobial therapy	Preauthorization requirements, audit and feedback, local guideline development	Antifungals	DOT per 1000 PD	22	17.1	*< .001*
						DOT per 1000 PD per quarter (slope)	− 0.5	− 0.1	0.22
					Echinocandins	DOT per 1000 PD	2.8	2.4	0.38
						DOT per 1000 PD per quarter (slope)	− 0.1	− 0.1	0.87
					Fluconazole	DOT per 1000 PD	19.2	14.8	*< .001*
						DOT per 1000 PD per quarter (slope)	− 0.5	0	*0.03*
Hurst et al. [30]	Quasi-experimental	444-bed tertiary care academic pediatric hospital	All inpatients prescribed antimicrobials	In-person feedback, no restrictions or preauthorization	Antifungals	DOT per 1000 PD (hematology, oncology)	874	640	*< 0.01*
						DOT per 1000 PD per month (slope)	5.4	0.7	> 0.05
Siegfried et al. [31]	Quasi-experimental	725-bed academic tertiary care center	Patients taking restricted antimicrobials	Stewardship pharmacist on weekends	Micafungin	DOT per 1000 PD	16.4	10.3	*0.04*
Nguyen-Ha et al. [32]	Quasi-experimental	315-bed pediatric hospital	Patients on day 3 of caspofungin therapy	72-h audits	Caspofungin	Mean start rate per 1000 patients	8	18.7	*< 0.001*
						Drug starts per 1000 patients per year (slope)	19.1	− 0.6	*0.001*
						Mean use rate per 1000 PD	14	29.3	*0.003*
						Drug use per 1000 PD per year (slope)	22.1	0.5	*0.014*
Guarascio et al. [33]	Matched control evaluation	450-bed university hospital	Adult ICU patients who received caspofungin	Care bundle for patients prescribed caspofungin	Caspofungin	Median days of caspofungin therapy	4.00	2.00	*0.001*
Di Pentima et al. [34]	Quasi-experimental	180-bed tertiary care academic pediatric hospital	Pediatric patients who received antibiotics in previous 24 h	Audit and feedback, preauthorization requirements	Azoles	Doses per 1000 PD	152 (24%)	137 (21.6%)	0.114
					Liposomal amphotericin	Doses per 1000 PD	14 (37.8%)	3 (8.1%)	*0.0003*
					Targeted antifungals^a^	Doses per 1000 PD	166 (24.8%)	141 (21%)	*0.02*
					Non-targeted antifungals^b^	Doses per 1000 PD	50	4	*< .0001*

*DDD* defined daily doses, *DOT* days of therapy, *PD* patient days

^a^Targeted antifungals = amphotericin B lipid formulations, fluconazole, voriconazole

^b^Non-targeted antifungals = amphotericin B deoxycholate, itraconazole

All studies were single-centered and quasi experimental in design with the earliest publication in 2001. Any data that did not pertain to outcomes of interest or antifungal agents were not included in the review. The fives studies that included clinical and performance outcomes had a study duration of 1–2 years and recorded data on 411 patients, the majority with a diagnosis of *Candida* infections. The eight studies used to reference antifungal consumption had an average of 4 years study duration, where each had a statistically significant decrease in antifungal use.

### Interventions

Intervention type and implementation varied across studies (Table 1). The five studies that evaluated AFS interventions included: implementation of a care bundle (1 study) [22], AFS pharmacist recommendations (1 study) [23], and development of a diagnostic tool (3 studies) [24–26]. Of the eight studies that evaluated general antimicrobial stewardship, many programs implemented multiple interventions. These included audit and feedback (5 studies) [27, 29, 30, 32, 34], preauthorization requirements or restriction (3 studies) [28, 29, 34], local guideline development (1 study) [29], care bundle development (1 study) [33], in-person feedback with no restrictions or preauthorization requirements (1 study) [30], and pharmacist stewardship coverage on weekends (1 study) [31].

### Performance outcomes

#### Appropriate antifungal choice

Two studies evaluated appropriate choice of antifungal [22, 23]. A higher percentage of patients were given the appropriate choice of antifungal in both studies. In one study, appropriate therapy after sensitivity testing was significantly higher in intervention vs. non-intervention groups, though rate of appropriate empiric therapy was unchanged. In the other study, rate of effective choice of antifungal was increased in the intervention vs. non-intervention group.

#### Time to therapy

Evaluation of time to antifungal therapy varied across five studies (Table 1). Four studies reported mean or median time to therapy in hours [23–26], and two studies reported percentages of patients receiving therapy within a specified timeframe [22, 24]. Time to therapy was improved in two of five studies. In one study, median time from Gram stain to effective antifungal hang time and order placement was significantly decreased, and in one study mean time to targeted therapy was decreased. The percentage of patients who received therapy within a timeframe was unchanged in two of two studies.

#### Antifungal consumption

Of the eight studies that evaluated general antimicrobial stewardship interventions, all reported data on antifungal consumption (Table 2). Various units were used to describe antifungal consumption including defined daily doses per 1000 patient-days or per 100 admissions (2 studies) [27, 28], days of therapy per 1000 patient-days (3 studies) [29–31], mean drug start and use rates (1 study) [32], median days of therapy (1 study) [33], and doses per 1000 patient-days (1 study) [34]. Due to the lack of common units, a direct quantitative comparison between studies was not possible. However, all studies reported either a significant decrease in use (7 of 8) or blunting of upward trend in use (1 of 8) of antifungal agents. Five studies evaluated all antifungal agents, and consumption was decreased in all five studies. Two studies evaluated echinocandins as a class, and consumption was unchanged in both studies. Caspofungin use was decreased in two studies, and micafungin use was decreased in one of one study. Consumption of azoles as a class was unchanged in one of one study, and fluconazole use was significantly decreased in one of two studies. Liposomal amphotericin B utilization was significantly decreased in one of one study.

### Clinical outcomes

#### Mortality

Four of five studies that evaluated AFS interventions reported data on mortality outcomes (Table 1) [22–26]. Of these, three reported in-hospital mortality and one reported 30-day mortality. All four studies found no significant change in mortality between intervention vs. non-intervention groups. In-hospital mortality occurred at a rate of 27% (32/118) in intervention groups and 23% (40/173) in non-intervention groups across three studies. In one study, 30-day mortality was 18% (3/17) in the intervention group and 33% (6/18) in the non-intervention group.

#### Hospital length of stay

All five studies that evaluated AFS interventions reported hospital length of stay (LOS) outcomes (Table 1). Overall LOS was unchanged across five of five studies and ranged from 9 to 25 days in intervention groups vs. 11–22 days in non-intervention groups. Intensive care unit LOS was also unchanged across two of five studies (range 0–10 vs. 0–15 days) [25, 26]. One study reported infection-related LOS (11 vs. 10 days), which was not significantly changed by the intervention [23].

## Discussion

Antifungal stewardship is an important component of a stewardship program given the rise in antifungal resistance and associated poor clinical outcomes [9, 18]. This is especially pertinent given the recent recognition and challenges associated with multi-drug resistant *C. auris* [12]. In order to properly combat antifungal resistance, additional AFS strategies and programs will be necessary.

The stewardship interventions varied across studies, but common stewardship interventions were used including audit and feedback or preauthorization requirements [27–29, 34]. Three studies were based on the introduction of a diagnostic tool for *Candida* species [24–26]. Similar to the IDSA recommendations for ASPs, the core members of an AFS team should include an infectious diseases specialist, clinical pharmacist, and a clinical microbiologist [3, 19]. Pharmacist recommendations were used as the primary intervention in two studies, which has been shown to improve care and clinical outcomes within ASPs [23, 31, 35]. Within the included AFS studies, all five articles reported including an infectious diseases-trained physician and a pharmacist as core members of the AFS programs. Microbiology was included in all studies, although it was unclear whether a clinical microbiologist was one of the core members of the AFS program. The formation of a multidisciplinary team with the necessary expertise will be key to the development and success of any AFS program [19].

Antifungal consumption was the most common outcome measure reported. Various approaches were used to describe consumption including defined daily doses, days of therapy, and dose adjusted to hospital bed occupancy. The use of antifungal days of therapy is the preferred metric according to IDSA guidelines as it is not impacted by dose adjustments and can be used in pediatrics where dosing is based on patient weight [3]. Notwithstanding the antifungal consumption metric, all studies reported decreased in antifungal use or blunting a previous upward trend in utilization. These decreases were apparent in studies reporting both overall antifungal utilization and those focusing on specific antifungal classes or drugs. Although it is clear that AFS can have a positive impact on antifungal consumption, the prescribing quality within these studies is not as clear. Only two studies evaluated whether appropriate antifungal therapy was prescribed. Both studies reported a higher percentage of patients given appropriate choices of antifungal therapy following implementation of an AFS intervention. The majority of studies did not evaluate appropriateness of antifungal prescribing as a process outcome. Previous research has shown a high proportion of inappropriate antifungal agent use including inadequate dosages or indications [14, 15, 36]. Given the overtreatment with antifungal therapy coupled with the rise in resistance, there should be greater focus on compliance with guideline recommendations as a reported performance measure.

Establishing the impact of AFS interventions on clinical outcomes should be a primary focus along with reporting antifungal utilization and other process outcomes. Only a few studies evaluated clinical outcomes including in-hospital or 30-day mortality and overall hospital length of stay. Notably, no significant change was reported in these clinical outcomes following the implementation of an AFS program. A meta-analysis of the implementation of hospital-based ASPs also found no difference in mortality following program implementation [37]. ASPs were associated with a significant decrease in hospital length of stay; however, these findings were based on only four studies [37]. At the very least, our findings support previous reports that stewardship programs do not adversely affect the level of patient care by focusing antifungal therapy on patients who really need it. However, similar to antimicrobial stewardship, AFS programs will need to evaluate clinical outcomes and show improvements in care in order to justify additional resources beyond the cost savings associated with decreased antifungal consumption.

Our study should be interpreted in view of certain limitations. The major one is the scarcity of literature and evidence to support AFS programs. Studies focusing on AFS programs were primarily published after 2010, which is consistent with the emergence of this concept [38]. Another important limitation is that all included studies were non-randomized and were primarily single center, quasi-experimental designs. Further, specific conclusions were drawn from studies with small numbers of patients. In addition, we focused our search on studies within the United States, which may limit the generalizability of our findings. Given the differences in healthcare around the world, our focus was to better understand the impact of AFS programs within the US healthcare system.

## Conclusion

Even though there is limited evidence on AFS programs and the interventions are highly variable, the evidence suggests that AFS effectively improves performance measures and decreases antifungal consumption. As an emerging field, AFS is similar to established ASPs, yet with different clinical priorities. Central to AFS expansion will be a standardized approach for the inclusion of core members within an AFS multidisciplinary team as well as comprehensively evaluating the quantity and quality of antifungal prescribing. Long-term evaluation is necessary to show the effect of AFS on patient and economic outcomes including mortality. Furthermore, as additional AFS studies become available, the development of guidelines will be necessary to benchmark best practices.

## Data Availability

All relevant data included within the paper were obtained from articles available in Pubmed and EMBASE using the search strategy described in the paper.
